# There is no such thing as a validated prediction model

**DOI:** 10.1186/s12916-023-02779-w

**Published:** 2023-02-24

**Authors:** Ben Van Calster, Ewout W. Steyerberg, Laure Wynants, Maarten van Smeden

**Affiliations:** 1 Department of Development and Regeneration, KU Leuven, Leuven, Belgium; 2 EPI-Center, KU Leuven, Leuven, Belgium; 3 Department of Biomedical Data Sciences, Leiden University Medical Center, Leiden, Netherlands; 4 Department of Epidemiology, CAPHRI Care and Public Health Research Institute, Maastricht University, Maastricht, Netherlands; 5 Julius Center for Health Sciences and Primary Care, University Medical Center Utrecht, Utrecht University, Universiteitsweg 100, 3584 CG Utrecht, Netherlands

**Keywords:** Risk prediction models, Predictive analytics, Internal validation, External validation, Heterogeneity, Model performance, Calibration, Discrimination

## Abstract

**Background:**

Clinical prediction models should be validated before implementation in clinical practice. But is favorable performance at internal validation or one external validation sufficient to claim that a prediction model works well in the intended clinical context?

**Main body:**

We argue to the contrary because (1) patient populations vary, (2) measurement procedures vary, and (3) populations and measurements change over time. Hence, we have to expect heterogeneity in model performance between locations and settings, and across time. It follows that prediction models are never truly validated. This does not imply that validation is not important. Rather, the current focus on developing new models should shift to a focus on more extensive, well-conducted, and well-reported validation studies of promising models.

**Conclusion:**

Principled validation strategies are needed to understand and quantify heterogeneity, monitor performance over time, and update prediction models when appropriate. Such strategies will help to ensure that prediction models stay up-to-date and safe to support clinical decision-making.

## Background

Clinical prediction models combine multiple patient and disease characteristics to estimate diagnostic or prognostic outcomes. Such models emerge continuously across a broad range of medical fields, often with the goal to guide patient risk stratification and to assist in making optimal decisions for individual patients. Prediction models need validation before implementation in clinical practice [1–3]. *Internal validation *refers to the validation of the model on the same patient population on which it has been developed, for example using a train-test split, cross-validation, or bootstrapping [4]. Conversely, *external validation *refers to the validation of the model on a new set of patients, usually collected at the same location at a different point in time (temporal validation) or collected at a different location (geographic validation) [5, 6].

Whereas internal validation focuses on reproducibility and overfitting, external validation focuses on transportability. Although assessing transportability of model performance is vital, an external validation with favorable performance does not prove universal applicability and does not justify the claim that the model is ‘externally valid’. Instead, the aim should be to assess performance across many locations and over time, in order to maximize the understanding of model transportability. Nevertheless, we argue that it is impossible to definitively claim that a model is ‘externally valid’, and that such terminology should be avoided. We discuss three reasons for this argument.

## Reason 1: patient populations vary

### Description

When validating a prediction model on an external dataset, patient characteristics are likely to be different than the characteristics of patients used for model development, even if patients in the validation dataset satisfy the same inclusion and exclusion criteria. Healthcare systems include different types of hospitals or practices, and healthcare systems vary between countries or even regions. Therefore, notable differences in patient characteristics (such as demographics, risk factors, and disease severity) between centers with similar inclusion and exclusion criteria are the rule rather than the exception [7, 8]. Such differences tend to be larger between different types of centers (e.g., secondary versus tertiary care hospitals), or if the validation data uses different inclusion and exclusion criteria. A prediction model that was developed in a tertiary care hospital may yield risk estimates that are invalid for the typical population seen at a regional hospital, or even for tertiary care hospitals in another country [9].

Patient characteristics may not only vary on average, but also in their distribution. Populations with more homogeneous distributions (i.e., less dispersed) tend to have lower discrimination performance as measured for example by the c-statistic or area under the receiver operating characteristic curve. This is because in populations where patients are more alike, estimated risks from the model will also be more alike: it becomes harder to separate those at higher risk from those at lower risk [10].

Besides the discriminative performance, the calibration performance is a key factor in the validity of prediction models. Calibration refers to the agreement between estimated risks from the prediction model and the corresponding observed proportions of events. A common miscalibration situation is that these estimated risks are too high or too low on average (poor “calibration in the large”). Furthermore, estimated risks may be too extreme (too close to 0 or 1) or not extreme enough (too far away from 0 or 1) compared to observed proportions [9]. Miscalibration can be detrimental to the medical decisions that are based on clinical prediction models [11, 12]. For example, if you would like to suggest biopsy when the risk of high-grade prostate cancer is at least 10%, you will perform many unnecessary biopsies when using a risk model that overestimates the risk. Hence, poor calibration is the Achilles heel for applicability of prediction models [9].

### Examples

**Fig. 1:**
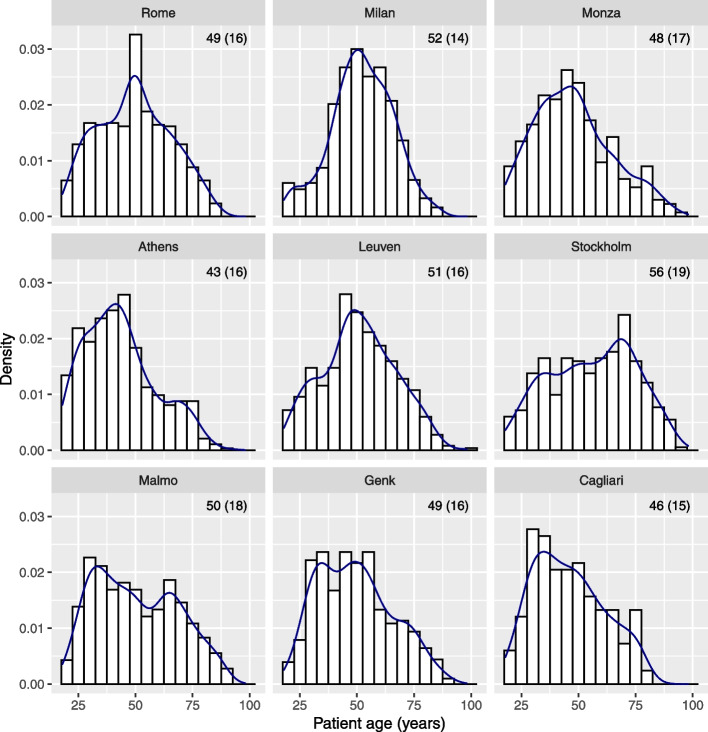
Distribution of patient age in the 9 largest centers from the ovarian cancer study. Histograms, density estimates, and mean (standard deviation) are given per center

**Fig. 2:**
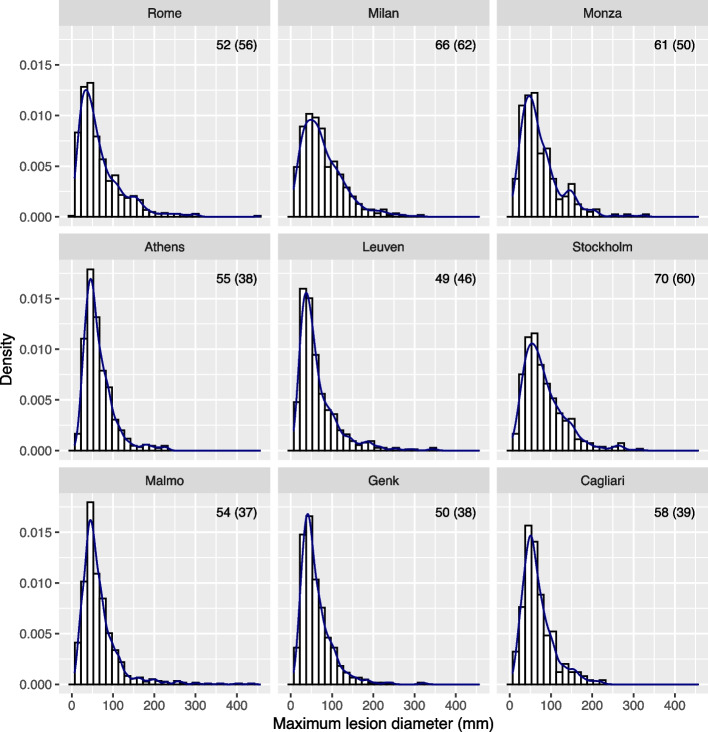
Distribution of maximum lesion diameter in the 9 largest centers from the ovarian cancer study. Histograms, density estimates, and median (interquartile range) are given per center

A recent multicenter cohort study externally validated prediction models to diagnose ovarian cancer [13]. Patients with an ovarian tumor were recruited at 17 centers in 7 countries. Participating sites were classified as oncology centers (gynecologic oncology unit within a tertiary center) versus other centers. The mean patient age in the 9 largest centers (*n* ≥ 166) varied between 43 and 56 years, and the standard deviation varied between 14 and 19 years (Fig. 1). The median maximum lesion diameter varied between 49 and 70 mm; the interquartile range varied between 38 and 62 mm (Fig. 2). If we focus at oncology centers in Italy (top row in Figs. 1 and 2) in order to compare similar centers from the same country, we still observe different distributions for these variables. Across the whole study sample, 26% of patients at oncology centers had a malignant tumor versus 10% at other centers. All models had higher c-statistics in oncology centers (c-statistics varied between 0.90 and 0.95) versus other centers (0.85 and 0.93).

The Wang clinical model for in-hospital mortality in coronavirus disease 2019 patients was validated using individual participant data from 24 cohorts covering 16 countries [14]. Median cohort size was 283 (range 25 to 25,056), mean patient age varied between 45 and 71 years, the percentage of male patients varied between 45 and 74%. Pooled performance estimates were 0.77 for the c-statistic, 0.65 for the observed over expected (O:E) ratio, and 0.50 for the calibration slope. The O:E ratio < 1 suggests that the model tends to overestimate the risk of in-hospital mortality. The calibration slope < 1 suggests that risk estimates also tend to be too extreme (i.e., too close to 0 or 1). Large heterogeneity in performance was observed, with 95% prediction intervals of 0.63 to 0.87 for the c-statistic, of 0.23 to 1.89 for the O:E ratio, and of 0.34 to 0.66 for the calibration slope. 95% prediction intervals indicate the performance that can be expected when evaluating the model in new clusters.

An external validation study of 104 prediction models for cardiovascular disease reported a median c-statistic of 0.76 for the models in their development data, compared to 0.64 at external validation [12]. When adjusting for differences in patient characteristics, the median c-statistic increased to 0.68. This suggests that about one third of the decrease in discrimination at external validation was due to more homogenous patient samples. This might be expected, given that clinical trial datasets were used for external validation, which often contain more homogeneous samples than observational datasets.

## Reason 2: measurements of predictors or outcomes vary

### Description

Predictor and outcome measurements or definitions may vary for various reasons, distorting their meaning in a model. First, measurements may be done using equipment from different manufacturers, with different specifications and characteristics. Typical examples are assay kits to quantify biomarker expression, or scanners used to obtain medical images. Second, measurements may depend on a specific method or timing, such as the measurement of blood pressure. Third, measurements may contain high degrees of subjectivity, such that the experience and background of the clinician plays a prominent role. This may cause variable model performance depending on the individual doing the observation. Fourth, biomarker measurements may contain intra-assay variation, analytical variation, and within-subject biological variation (including cyclical rhythms) [15, 16]. Fifth, clinical practice patterns, such as the timing and type of medication or laboratory test orders, tend to vary between clinicians and geographical locations [17, 18]. Such measurements are increasingly used in prediction modeling studies based on electronic health records.

Such heterogeneity in measurement procedures will affect model performance [19, 20]. Depending on how these measurements differ between development and validation, the discriminative performance and in particular the calibration performance can be severely affected. In contrast to intuition, “better” measurements at validation, e.g., predictors measured under stricter protocols than in the development data, may not lead to improved, but instead to deteriorated performance of the prediction model [19, 20].

### Examples

Using 17,587 hip radiographs collected from 6768 patients at multiple sites, a deep learning model was trained to predict hip fracture [21]. The c-statistic on the test set (5970 radiographs from 2256 patients; random train-test split) was 0.78. When non-fracture and fracture test set cases were matched on patient variables (age, gender, body mass index, recent fall, and pain), the c-statistic for hip fracture decreased to 0.67. When matching also included hospital process variables (including scanner model, scanner manufacturer, and order priority), the c-statistic for hip fracture was 0.52. This suggests that variables such as the type of scanner can inflate predictions for hip fracture.

The Wells score calculates the pretest probability of pulmonary embolism in patients suspected to have the condition [22]. A variable in the model is “an alternative diagnosis is less likely than pulmonary embolism”. This variable is subjective, and is likely to have interobserver variability. Studies have indeed reported low kappa values for the Wells score (0.38, 0.47) and for the abovementioned subjective variable on its own (0.50) [23, 24].

A systematic review of prognostic models for delirium reported considerable variation in delirium assessment method and frequency across the 27 included studies [25]. Reported methods included the Confusion Assessment Method (CAM), short CAM, Family CAM, Delirium Rating Scale Revised 98, Nursing Delirium Screening Scale, Delirium Assessment Scale, Memorial Delirium Assessment Scale, Delirium Symptom Interview, ward nurse observation, and retrospective chart review. Frequency varied between once to more than once per day. As a result, delirium incidence varied widely.

Seven expert radiologists were asked to label 100 chest x-ray images for the presence of pneumonia [26]. These images were randomly selected after stratification by classification given by a deep learning model (50 images labeled as positive for pneumonia, 50 labeled as negative). There was a complete agreement for 52 cases, 1 deviating label for 24 cases, 2 deviating labels for 13 cases, and 3 deviating labels for 11 experts. Pairwise kappa statistics varied between 0.38 and 0.80, with a median of 0.59.

Wynants and colleagues evaluated the demographic and ultrasound measurements obtained from 2407 patients with an ovarian tumor that underwent surgery [27]. Each patient was examined by one of 40 different clinicians across 19 hospitals. The researchers calculated the proportion of the variance in the measurements that is attributable to systematic differences between clinicians, after correcting for tumor histology. For the binary variable indicating whether the patient was using hormonal therapy, the analysis suggested that 20% of the variability was attributed to the clinician doing the assessment. The percentage of patients reporting the use of hormonal therapy roughly varied between 0 and 20%. A subsequent survey among clinicians revealed that clinicians reporting high rates of hormonal therapy had assessed this more thoroughly, and that there was a disagreement of the definition of hormonal therapy.

In a retrospective study, 8 radiologists scored four binary magnetic resonance imaging (MRI) features that are predictive of microvascular invasion (MVI) on MRI scans of 100 patients with hepatocellular carcinoma [28]. In addition, the radiologists evaluated the risk of MVI on a five-point scale (definitely positive, probably positive, indeterminate, probably negative, definitely negative). Kappa values were between 0.42 and 0.47 for the features, and 0.24 for the risk of MVI. The c-statistic of the risk for MVI (with histopathology as the reference standard), varied between 0.60 and 0.74.

## Reason 3: populations and measurements change over time

### Description

Every prediction model is subject to an — usually implicit — expiration date [29]. In the fast-changing and developing world of medicine, patient populations, standards of care, available treatment options, and patient preferences, measurement and data registration procedures change over time [30]. Also, baseline risks for conditions are expected to change over time, for instance, because patient populations tend to become older due to longer life expectancies, or due to shifts in life style and dietary patterns, and the availability of more effective and tailored preventive measures and information. These changes in population characteristics over time are to be expected and may cause performance drifts of prediction models. For example, calibration drift has been well documented [31]. It is therefore increasingly recognized that prediction models need to be updated regularly [32].

A particularly difficult topic is that implementing a prognostic prediction model in clinical practice may invalidate model predictions [33]. The implementations of risk models often aim to identify patients in which interventions are most beneficial. If the implementation of the model leads to effective interventions in high-risk patients, events will be prevented in a proportion of patients. The predictions of the model were derived under the absence of model-induced interventions, and may no longer be accurate; we never observe what could have happened without intervention. In addition, implementation of the model may improve the quality of the measurements of variables that are included as predictors in the model [34]. This should be beneficial as such, but the validity of predictions may be distorted.

### Examples

Davis and colleagues developed prediction models for hospital-acquired acute kidney injury using data from patients who were admitted to Department of Veterans Affairs hospitals in the United States in 2003 [35]. The models were developed using different algorithms (e.g., logistic regression, random forest, neural networks), and were validated over time using similar data from patients admitted up to and including 2012. Although discrimination remained fairly stable, with c-statistics roughly around 0.75, there was clear evidence of calibration drift for all models: the risk of the event became increasingly overestimated over time. Accompanying shifts in the patient population were noted: for example, the incidence of the event steadily decreased from 7.7 to 6.2%, age at admission increased, the proportion of patients with a history of cancer or diabetes increased, and the use of various medications increased.

EuroSCORE is a model that predicts in-hospital mortality for patients undergoing cardiac surgery [36]. Using data on 317,292 cardiac surgeries performed in Great Britain and Ireland between 2001 and 2011, it was observed that EuroSCORE overestimated the risk of in-hospital mortality, and that the overestimation aggravated over time [36]. In the beginning of the study period, observed mortality was 4.1% whereas EuroSCORE had an average estimated risk of 5.6%. At the end, observed mortality was 2.8% but the average estimated risk was 7.6%. The c-statistic showed no systematic deterioration, with values varying between 0.79 and 0.85. Furthermore, temporal changes were observed for several predictors (e.g., average age and prevalence of recent myocardial infarction increased) and surgical procedures (e.g., fewer isolated coronary artery bypass graft procedures). The authors further stated that surgeons may have been more willing to operate on patients due to improvements in anesthetic, surgical, and postoperative care.

## Conclusions

We presented three reasons why prediction models are never truly validated. A single external validation study in a specific geographical location, in a single time frame, for a sample from a specific patient population is only a snapshot. Such a single study may provide relevant information about the performance of the prediction model in a specific setting with a particular measurement and time context, but cannot claim transportability beyond that setting. Based on such a study, it is inappropriate to conclude whether a model has been successfully ‘validated’. In addition, claims about validity are often based on simplistic criteria using the c-statistic as a measure of discrimination. For example, a model may be declared “validated” if the 95% confidence interval of the c-statistic at validation includes the point estimate of the c-statistic that was originally reported, or if the obtained point estimate of the c-statistic exceeds a certain target value, such as > 0.7 or > 0.8 [37, 38]. Such criteria lack scientific underpinning.

The current focus on developing new models should shift to a focus on more extensive, well-conducted, and well-reported validation studies of promising models. We advise to embrace heterogeneity at model development and at external validation and provide the following general recommendations [10, 39–45].

1. When developing a prediction model, consider the inclusion of multiple settings/locations such as by conducting a multicenter or an individual participant data study [40–42, 45]. Where possible, (a) quantify performance heterogeneity using internal–external cross-validation procedure, where each study is left out once [3, 46]; (b) standardize predictor variables in terms of definition or measurement protocol to reduce prediction measurement heterogeneity; (c) investigate operator-induced measurement variability [27, 47]; and (d) consider to include an operational or population-level characteristic as a predictor (e.g., type of center [45]).
2. When validating a prediction model, inclusion of multiple settings or locations allows to study performance heterogeneity across settings [10, 39, 43, 44].
3. Use appropriate statistical methodology and sample size for model development and/or validation studies, and report fully and transparently [48]. Follow the TRIPOD reporting guideline (Transparent Reporting of a multivariable prediction model for Individual Prognosis Or Diagnosis), including the newly available TRIPOD-Cluster extension where appropriate [49–52]. For example, all predictors should be defined, and the model itself should be made available to allow independent external validation studies [3].
4. Before implementing a model in a specific location, it is recommended to conduct a local validation study. Consider to monitor performance over time and to (dynamically) update the model, in particular when calibration is problematic [31, 32, 53].

We question the requirement from some journals that model development studies should include “an external validation”. Apart from the arguments presented above, this requirement may induce selective reporting of a favorable result in a single setting. But is it never good enough? Imagine a model that has been externally validated in tens of locations, representing a wide range of settings, using recent data. Discrimination and calibration results were good, with limited heterogeneity between locations. This would obviously be an important and reassuring finding. Even then, there is still no 100% guarantee that the prediction model will also work fine in a new location. Moreover, it remains unclear how populations change in the future.

In practice, calibration is typically more vulnerable to geographic and temporal heterogeneity than discrimination [9, 12–14, 20, 35, 36, 44]. We stress that calibration assessment in the external validation sample is at least as important as discrimination [9]. If a calibration curve with a narrow 95% confidence interval is close to the ideal diagonal line, one may conclude that risk estimates were appropriate at least for the specific context of the external validation study. For any performance criterion, a meaningful evaluation requires a sufficient sample size. Rules of thumb suggest that at least 100 to 200 cases in the smallest outcome category are required for external validation studies [54, 55]. More refined sample size procedures for model validation have been proposed recently [48].

In conclusion, clinical prediction models are never truly validated due to expected heterogeneity in model performance between locations and settings, and over time. This calls for a stronger focus on validation studies, using principled validation strategies to quantify heterogeneity, regularly monitor model performance, and update models [31, 32]. Such strategies help to ensure that prediction models stay up-to-date to support medical decision-making.

## Data Availability

Information from all examples (except the example on ovarian cancer) was based on information available in published manuscripts. The data used for the ovarian cancer example were not generated in the context of this study and were reused to describe differences between populations from different centers. The data cannot be shared for ethical/privacy reasons.

## Abbreviations

O:E: Observed over expected
CAM: Confusion Assessment Method
MVI: Microvascular invasion
MRI: Magnetic resonance imaging
TRIPOD: Transparent Reporting of a multivariable prediction model for Individual Prognosis Or Diagnosis
